# Molecular and Cellular Effects of Chemical Chaperone—TUDCA on ER-Stressed NHAC-kn Human Articular Chondrocytes Cultured in Normoxic and Hypoxic Conditions

**DOI:** 10.3390/molecules26040878

**Published:** 2021-02-07

**Authors:** Magdalena Kusaczuk, Monika Naumowicz, Rafał Krętowski, Bartosz Cukierman, Marzanna Cechowska-Pasko

**Affiliations:** 1 Department of Pharmaceutical Biochemistry, Medical University of Bialystok, Mickiewicza 2A, 15-222 Bialystok, Poland; r.kretowski@umb.edu.pl (R.K.); mapasko@gmail.com (M.C.-P.); 2 Faculty of Chemistry, University in Bialystok, K. Ciołkowskiego 1K, 15-245 Bialystok, Poland; monikan@uwb.edu.pl; 3 Department of Orthopedics and Traumatology, Regional Hospital in Sokolka, Sikorskiego 40, 16-100 Sokolka, Poland; bartoszcukierman@gmail.com

**Keywords:** chondrocytes, ER stress, inflammation, tauroursodeoxycholate, osteoarthritis

## Abstract

Osteoarthritis (OA) is considered one of the most common arthritic diseases characterized by progressive degradation and abnormal remodeling of articular cartilage. Potential therapeutics for OA aim at restoring proper chondrocyte functioning and inhibiting apoptosis. Previous studies have demonstrated that tauroursodeoxycholic acid (TUDCA) showed anti-inflammatory and anti-apoptotic activity in many models of various diseases, acting mainly via alleviation of endoplasmic reticulum (ER) stress. However, little is known about cytoprotective effects of TUDCA on chondrocyte cells. The present study was designed to evaluate potential effects of TUDCA on interleukin-1β (IL-1β) and tunicamycin (TNC)-stimulated NHAC-kn chondrocytes cultured in normoxic and hypoxic conditions. Our results showed that TUDCA alleviated ER stress in TNC-treated chondrocytes, as demonstrated by reduced CHOP expression; however, it was not effective enough to prevent apoptosis of NHAC-kn cells in either normoxia nor hypoxia. However, co-treatment with TUDCA alleviated inflammatory response induced by IL-1β, as shown by down regulation of *Il-1β*, *Il-6*, *Il-8* and *Cox2*, and increased the expression of antioxidant enzyme *Sod2*. Additionally, TUDCA enhanced *Col IIα* expression in IL-1β- and TNC-stimulated cells, but only in normoxic conditions. Altogether, these results suggest that although TUDCA may display chondoprotective potential in ER-stressed cells, further analyses are still necessary to fully confirm its possible recommendation as potential candidate in OA therapy.

## 1. Introduction

Osteoarthritis (OA) is a chronic disease that affects diarthrodial joints. This degenerative disorder involves pathology of all joint districts, including synovium, meniscus, bone, ligaments/tendons, and articular cartilage [1]. Over 150 million people worldwide are already affected by OA, and approximately 37% of the population of elderly people in the U.S. is projected to develop OA in the future [2,3]. Multiple risk factors, including obesity, age, prior joint injuries, and genetic factors, contribute to osteoarthritis development, which places OA as a disease with increasing incidence of occurrence in modern societies, but still with limited treatment options [4,5].

One of the joint structures heavily affected by progressing OA is articular cartilage. It is composed of chondrocyte cells, which are the only resident cell type in this structure. Chondrocytes are responsible for synthesis and turnover of extracellular matrix (ECM) components, making them a key regulators of growth, mechanical support, and proper functioning of diarthrodial joints [6,7]. One of the main features of cartilage degeneration was found to be apoptosis of chondrocyte cells [6,8]. Thus, loss of chondrocytes due to apoptotic cell death has been proposed as a possible mechanism of OA pathology [6]. Although apoptosis of chondrocytes was found to result mainly from activation of extrinsic and mitochondrial pathways [9,10], recently the endoplasmic reticulum (ER) stress-dependent pathway has been reported to be associated with chondrocyte death in many in vitro and animal model studies [11,12,13,14,15]. Yang et al. have found that ER stress evoked apoptosis of rat chondrocytes and down-regulated expression levels of ECM proteins, including type II collagen and aggrecan in articular cartilage [11]. Likewise, occurrence of ER stress has been demonstrated in chondrocytes of osteoarthritis cartilage in humans [12,13]. Increased levels of GRP78, PDI, CHOP, and caspase-12 have been detected in articular cartilage of OA patients, indicating the role of ER stress and ER stress-mediated apoptosis in OA progression [13,16]. Nevertheless, pathological events accompanying OA development are not the only ones causing ER stress in cartilage tissue. It is believed that normal chondrocytes might be naturally predisposed to develop and deal with ER stress [7]. Since adult cartilage is avascular, natural supplies of nutrients such as glucose and oxygen are very limited and may reach chondrocytes only by diffusion. This makes chondrocytes chronically exposed to nutrient deprivation and prone to ER stress induction [6,7,17,18]. Additionally, in the process of initiation and progression of OA, changes in normal chondrocytes have been demonstrated to occur not only due to physiological alterations and increased mechanical pressure, but also to result from the exposure to pro-inflammatory cytokines [19,20]. Indeed, increased expression of pro-inflammatory agents, especially interleukin-1β (IL-1β), in the serum and cartilage of OA suffering individuals was demonstrated to be involved in OA progression [21]. IL-1β may contribute to impairment of chondrocyte functioning in many ways, including decreased expression of cartilage-structural proteins, such as type II collagen and aggrecan and augmented production of catabolic enzymes, such as matrix metalloproteinases, which leads to cartilage destruction [22]. Moreover, a link between inflammation and ER stress exists. The three branches of the unfolded protein response (UPR) intersect with a multitude of stress and inflammatory signaling networks including the I kappa B kinase (IKK)- and JNK-AP-dependent pathways [23,24]. In this respect, IL-1α and IL-6 have been shown to up-regulate the expression of ER stress components and to further sensitize chondrocytes to IL-1β treatment [17,25,26,27]. Additionally, it has been demonstrated that stimulation with IL-1β caused overexpression of *Ire-1α* and *Perk* in OA chondrocytes, while IL-6 treatment resulted in attenuated expression of both transcripts [28].

Regarding the importance of ER functioning in chondrocytes, ER stress response has already attracted attention as a new growing area of investigations in cartilage biology in OA. To date, varying therapies including pharmacologic, nonpharmacologic, and surgical treatments have been applied in an effort to limit OA progression. Currently, several medications including non-steroidal anti-inflammatory drugs are routinely prescribed [5]. However, any specific treatment aimed at preventing or retarding cartilage degradation in OA has been developed, and all available treatments provide only short-term symptomatic pain relief. Conventional drugs for chronic pain, such as non-steroidal anti-inflammatory drugs, increase the risk of adverse effects, whereas the efficacy of oral dietary supplements containing hyaluronic acid (HA), glucosamine sulfates, and chondroitin sulfates is still questionable according to the clinical data [29,30,31]. In this respect, questing for the alternative therapies bringing relief to the suffering patients is still required. 

Lately, novel efficient approaches targeted at reducing ER stress in various cells have been in the spotlight. A group of pharmacological agents possessing potentially beneficial effects against ER stress are known as “chemical chaperones” [23,32]. Tauroursodeoxycholic acid (TUDCA) is one such compound that is best known for its chaperoning activity [32]. TUDCA is a naturally occurring hydrophilic bile acid, which is a taurine conjugate of ursodeoxycholic acid approved by FDA (US Food and Drug Administration) for treatment of certain cholestatic liver diseases [32]. TUDCA has been known to evoke beneficial effects in many in vitro and in vivo studies performed on models of a multitude of ER stress-related diseases such as diabetes, obesity, and neurodegenerative disorders [32]. In this respect, utilization of chemical chaperones as potential agents in OA treatment seems to be an interesting and promising avenue in contemporary pharmacology of joint diseases.

Although recent findings have demonstrated good efficiency of chemical chaperones in alleviating ER stress in broad spectrum of cell types, only a limited amount of data is available in terms of chemical chaperones function in chondrocytes [6,33]. Thus, in this study we investigated whether TUDCA was able to reduce ER stress, alleviate inflammation, restore collagen type II expression, and prevent apoptosis of human articular chondrocytes treated with IL-1β and tunicamycin (TNC). Additionally, the influence of HA on IL-1β-treated chondrocytes was evaluated in order to establish if HA could serve as a potential co-therapeutic agent for TUDCA in OA treatment. The pharmacological agents used in the study together with their previously reported activity in chondrocytes are listed in Table 1.

## 2. Results

### 2.1. Analysis of Cell Viability

**Figure 1:**
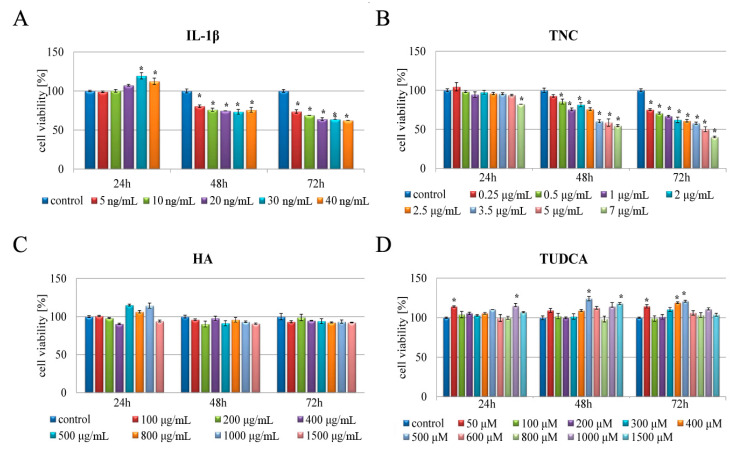
Effect of IL-1β, TNC, TUDCA, and HA on the viability of NHAC-kn chondrocytes. Chondrocytes were treated for 24, 48, and 72 h with the indicated concentrations of IL-1β (**A**), TNC (**B**), HA (**C**), and TUDCA (**D**) in normoxic conditions. The viability of chondrocytes was determined using the MTT assay. The results represent means for pooled triplicate values from three independent experiments. Significant changes are expressed relative to controls and marked with asterisks. Statistical significance was considered if * *p* < 0.05.

To investigate whether potentially cytotoxic (IL-1β, TNC) but also cytoprotective agents (TUDCA, HA) affect chondrocyte viability, cells were treated with various concentrations of IL-1β (5–40 ng/mL), TNC (0.25–7 µg/mL), TUDCA (50–1500 µM), and HA (100–1500 µg/mL) for 24, 48, and 72 h and subjected to MTT analysis (Figure 1). As expected, IL-1β and TNC reduced cell viability in a concentration- and time-dependent manner. IL-1β did not affect the viability of NHAC-kn cells after 24 h, but caused cytotoxic effect after 48 and 72 h, decreasing cell viability up to 62% (40 ng/mL, 72 h) (Figure 1A). Additionally, while TNC-treatment had no cytotoxic effect on chondrocytes at 24 h, it significantly decreased proliferation in a time-dependent manner over 48 and 72 h. The most pronounced effect was observed after 72 h, as 7 μg/mL TNC led to nearly 60% reduction in cell viability when compared to the controls (Figure 1B). Based on these results, representative concentrations of either IL-1β or TNC were chosen for further examinations. In order to mimic inflammation in cultured chondrocytes, the cells were further treated with 10 ng/mL of IL-1β, which represents an experimental model for inflammation in vitro [22,40,41,42,43]. Additionally, the concentration of 2.5 µg/mL of TNC was chosen as an inducer of ER stress, which is in line with previously published in vitro studies [6,18].

Among the range of HA concentrations, the influence on cell viability was mostly insignificant independently of the time and dose of applied HA, confirming that it is generally safe and non-toxic to cells (Figure 1C). Given this, a concentration of 800 µg/mL matching the range of achievable (0.5–4 mg/mL) HA concentrations in the synovial fluid of the diarthrodial joints, was examined in further experiments [34]. Similarly, TUDCA was not cytotoxic to chondrocyte cells, and even promoted cell viability in certain concentrations (500 and 1500 µM after 48 h and 400 and 500 µM after 72 h) (Figure 1D). Thus, a concentration of 300 µM was chosen in order to act as chemical chaperone, which is in general accordance with other reports [6,33]. In further examinations, the above specified concentrations of each compound were applied. 

### 2.2. Induction of Hypoxia, Cell Morphology, and Apoptosis

**Figure 2:**
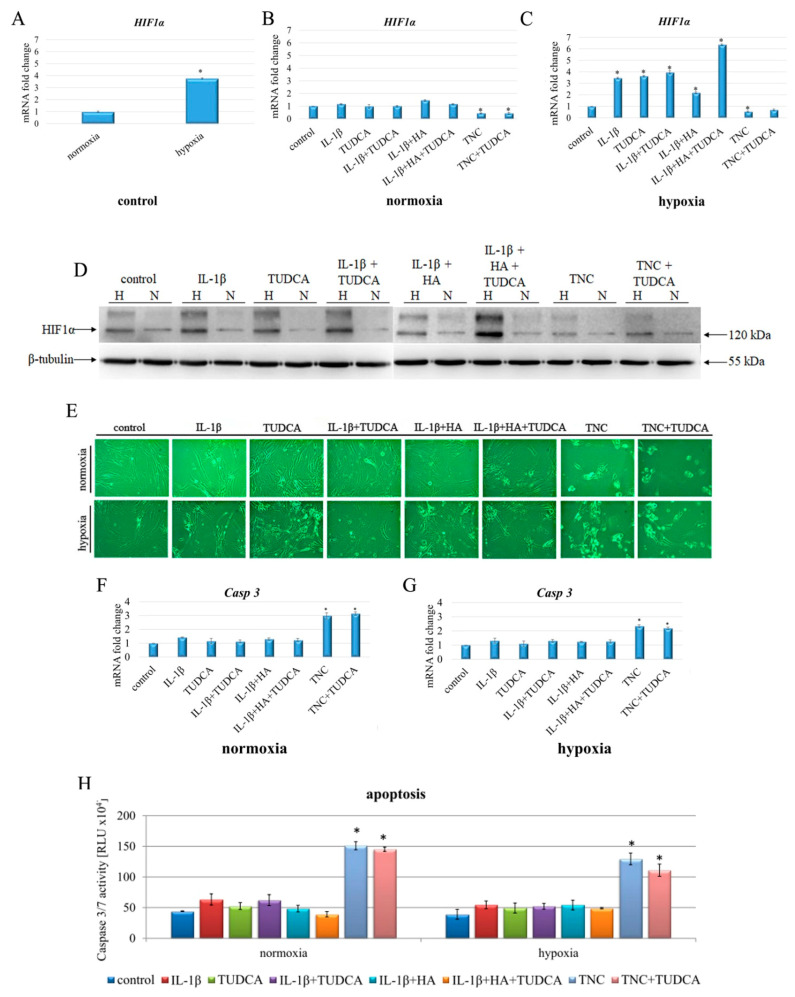
Characterization of hypoxia, cell morphology, and apoptosis in NHAC-kn chondrocyte cells stimulated with IL-1β, TUDCA, IL-1β + TUDCA, IL-1β + HA, IL-1β + HA + TUDCA, TNC, and TNC + TUDCA for 72 h. Relative quantification of *HIF1α* expression in normoxic vs. hypoxic control cells (**A**). Relative quantification of *HIF1α* expression in cells subjected to indicated treatments in normoxic (**B**) and hypoxic (**C**) conditions. Protein expression of HIF1α (**D**). Representative Western blot images are shown. β-tubulin was used as the loading control. Morphological characteristics of chondrocytes subjected to a variety of indicated treatments (**E**). Representative photographs evaluated by phase contrast microscopy are shown (magnification ×100). Relative quantification of *Casp 3* expression in normoxic (**F**) and hypoxic (**G**) conditions. Results of qRT-PCR are shown as relative fold change in mRNA expression in comparison to untreated controls, where expression level was set as 1. Caspase 3/7 activity in normoxic and hypoxic conditions (**H**). Results of qPCR and luminescence-based analyses are presented as mean ± SD from three independent experiments run in triplicate. Significant changes are expressed relative to controls and marked with asterisks. Statistical significance was considered if * *p* < 0.05.

The concentration of O_2_ in cartilage ranges from 5% in the upper zones of healthy cartilage and drops to even 1% close to the calcified cartilage, making chondrocytes particularly influenced by hypoxia inducible factor 1α (HIF1α) [50,51]. The centrality of HIF1α in cell adaptation to hypoxia makes it a strong candidate to be a marker of hypoxic cells [52]. In this respect, we performed our experiments in both normoxic (21% O_2_) and hypoxic (1% O_2_) conditions, first evaluating the expression of HIF1α in treated chondrocytes (Figure 2A–D). Although 20% of oxygen is unachievable in chondrocyte cells, and only 5–8% of O_2_ is normally present in these cells, using ≈20% O_2_ is commonly accepted model of normoxic conditions in plethora of different cell types [53]. Thus, it has also been translated into chondrocyte-based in vitro research as standard cell culture condition, called “normoxia’’ [54,55]. As shown by q-RT PCR and Western blot analyses, cells cultured in 1% O_2_ exhibited marked overexpression of HIF1α on both mRNA and protein levels (Figure 2A,D). The effect of particular treatment in cells cultured in normoxic conditions was almost undetectable (Figure 2B); however, results of HIF1α expression differed significantly between treatments in hypoxia cultured cells (Figure 2C). Chondrocytes treated with IL-1β + HA + TUDCA showed the most pronounced overexpression of HIF1α (over six-fold increase in comparison to control), while cells treated with TNC exhibited marked down-regulation of HIF1α (Figure 2C,D).

Microscopic observations were carried out to determine whether the applied treatments were accompanied by alterations in cellular morphology and reduced proliferation of NHAC-kn cells. The majority of applied treatments (IL-1β, TUDCA, IL-1β+TUDCA, IL-1β+HA, IL-1β+HA+TUDCA) did not visibly affect the proliferation and morphology of chondrocytes (Figure 2E). Cells showed undisrupted growth and preserved angular and adherent with rich cytoplasm (Figure 2E). However, treatment with TNC alone resulted in marked reduction in cell proliferation with altered-shaped, more rounded, and blebbed chondrocytes. Additionally, no visible signs of protective effect in TNC+TUDCA group were observed (Figure 2E). 

Next, using the luminescent assay and q-RT PCR, we assessed apoptotic cell death in treated chondrocytes (Figure 2F–H). To determine whether particular treatments affected chondrocytes’ apoptosis, caspase 3/7 activity and *Casp 3* expression was assessed (Figure 2F–H). Results of the qRT-PCR analysis revealed that in normoxic conditions, significant up-regulation of *Casp 3* occurred in cells treated with TNC and TNC+TUDCA-treated group (three- and 3.1-fold increase, respectively) (Figure 2F). Luminescent assay confirmed that TNC and TNC+TUDCA treatment caused significant elevation in caspase 3/7 activity, up to 3.8- and 3.7-fold increase in both treatments, indicating that TUDCA did not augment the survival of chondrocytes in TNC treated cultures (Figure 2H). A similar trend was observed in hypoxic conditions; however, here the ratios of *Casp 3* expression and caspase 3/7 activity were lower, reaching a 2.3- and 2.2-fold increase in *Casp 3* and a 3.3- and 2.8-fold increase in caspases activity, respectively, in TNC and TNC+TUDCA in comparison to control (Figure 2G,H).

### 2.3. Analysis of ER Stress

**Figure 3:**
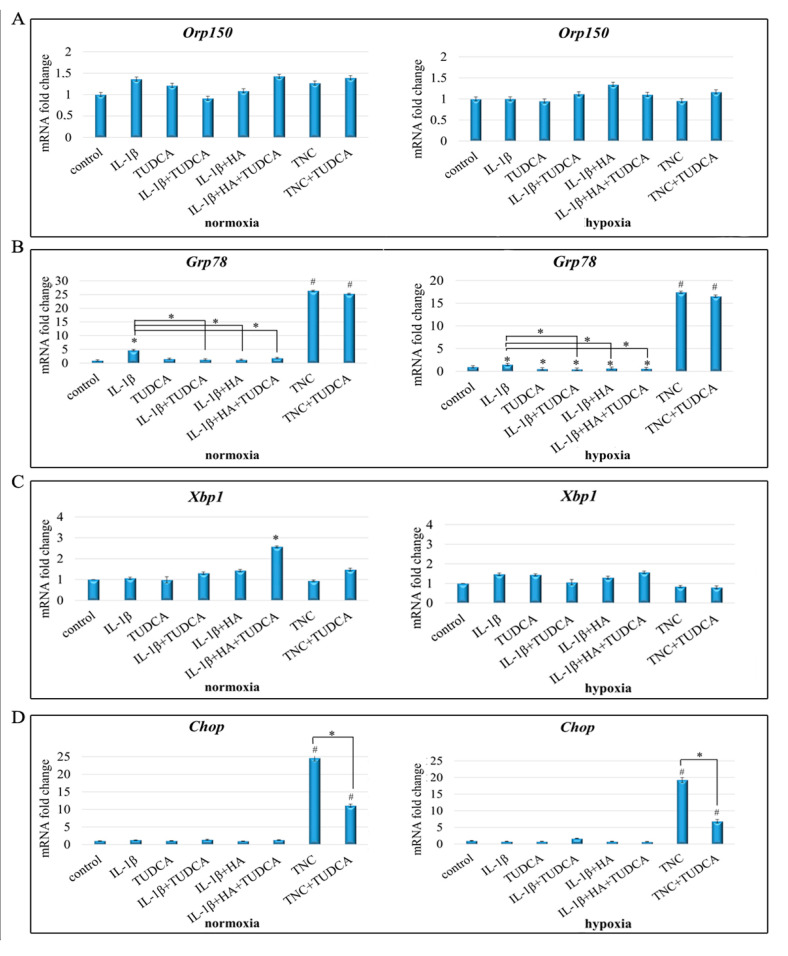

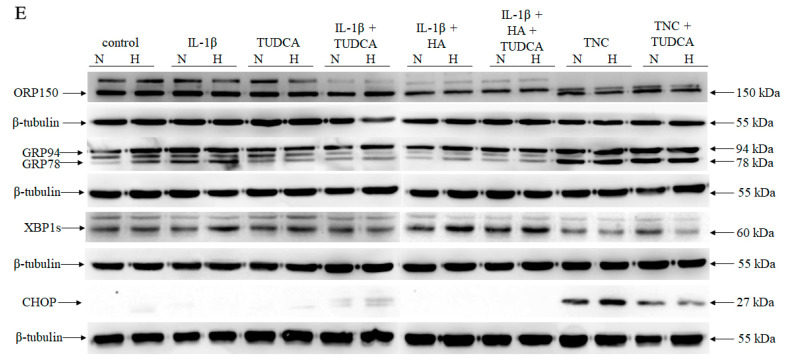
Expression of ER stress markers in NHAC-kn chondrocyte cells stimulated with IL-1β, TUDCA, IL-1β+TUDCA, IL-1β+HA, IL-1β+HA+TUDCA, TNC, and TNC+TUDCA for 72 h. Relative quantification of *Orp150* (**A**), *Grp78* (**B**), *Xbp1* (**C**), and *Chop* (**D**), expressions in normoxic and hypoxic conditions. Results of qRT-PCR are shown as relative fold change in mRNA expression in comparison to untreated controls, where expression level was set as 1. Protein expressions of ORP150, GRP78, GRP94, XBP1s, and CHOP (**E**). Representative Western blot images are shown. β-tubulin was used as the loading control. Results of qPCR analysis are presented as mean ± SD from three independent experiments run in triplicate. Significant changes are expressed relative to controls and marked with asterisks and hashes placed above the columns, or as specifically indicated with dashes linking compared treatments. Statistical significance was considered if * *p* < 0.05 or # *p* < 0.001.

To investigate the effect of indicated treatments (IL-1β, TUDCA, IL-1β+TUDCA, IL-1β+HA, IL-1β+HA+TUDCA, TNC and TNC+TUDCA) on ER stress in NHAC-kn chondrocytes cultured in normoxia and hypoxia, qRT-PCR (Figure 3A–D) and Western blot (Figure 3E) analyses were performed. In this respect, markers of ER stress such as molecular chaperones ORP150, GRP78, and GRP94, as well as transcription factors XBP1s and CHOP, were evaluated on mRNA and protein levels. Both proteomic and transcriptomic analyses showed that the expression of ORP150 did not alter significantly in most of the tested variants of treatments (IL-1β, TUDCA, IL-1β+TUDCA, IL-1β+HA, IL-1β+HA+TUDCA); however, Western blot results revealed that stimulation with TNC and TNC+TUDCA caused induction of the glycosylated form of ORP150 (protein with the molecular weight of 170 kDa), which indicates occurrence of TNC-induced ER stress. However, co-treatment with TUDCA was insufficient to alter the expression level of ORP150/170 ratio in either normoxic or hypoxic conditions (Figure 3E). Further, qRT-PCR analysis showed that in normoxic conditions *Grp78* was significantly up-regulated by IL-1β treatment and that this effect was markedly diminished in IL-1β+TUDCA, IL-1β+HA, and IL-1β+HA+TUDCA treated cells in comparison to IL-1β stimulation itself (Figure 3C). Similar results were observed in hypoxic cells (Figure 3C). Western blot analysis confirmed the mRNA results for GRP78 and additionally revealed that the expression level of GRP94 is also diminished in TUDCA-treated groups in comparison to IL-1β stimulation (Figure 3E). Interestingly, in both experimental conditions, GRP78/GRP94 were markedly overexpressed in TNC-treated cells, but this effect was not alleviated by TUDCA co-treatment (Figure 3B,E). Expression of pro-survival transcription factor *Xbp1* on mRNA was only slightly affected by the indicated treatments (Figure 3C). However, Western blot analysis of the spliced version of this protein showed that in normoxia XBP1s was overexpressed in IL-1β+HA+TUDCA-treated chondrocytes, whereas in hypoxia, overexpression of XBP1s was observed in IL-1β, IL-1β+HA, and IL-1β+HA+TUDCA and diminished expression was observed in TNC and TNC+TUDCA-treated cells in comparison to control (Figure 3E). The expression of pro-apoptotic *Chop* was significantly up-regulated only in TNC treated cells in both normoxic and hypoxic conditions (up to 15- and 20-fold up-regulation, respectively) (Figure 3D). Interestingly, co-treatment with TUDCA markedly down-regulated TNC-induced overexpression of Chop (Figure 3E). The results of CHOP expression on protein level confirmed qRT-PCR results (Figure 3E). Altogether, these results show that treatment with IL-1β and TNC result in deregulation of ER balance, and TUDCA occurred to be effective in diminishing the expression of CHOP—the pro-apoptotic mediator of the UPR signaling—suggesting potential benefits of TUDCA in restoring ER homeostasis in chondrocyte cells.

### 2.4. Analysis of Inflammation

**Figure 4:**
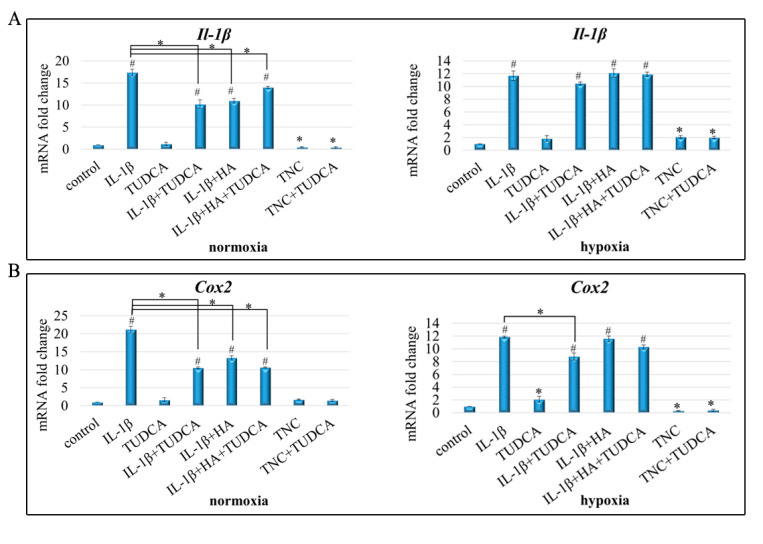

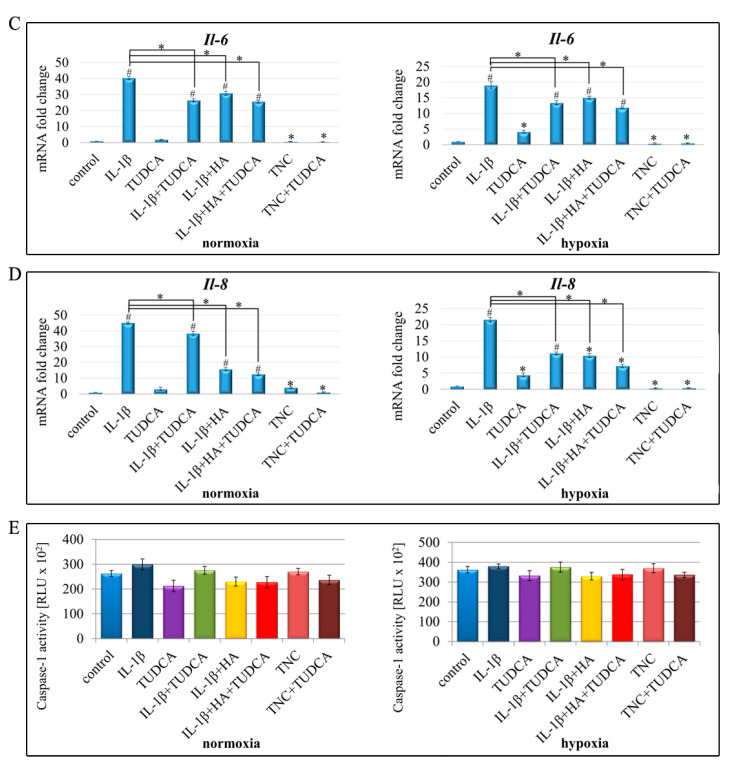
Inflammatory response in NHAC-kn chondrocyte cells stimulated with IL-1β, TUDCA, IL-1β+TUDCA, IL-1β+HA, IL-1β+HA+TUDCA, TNC, and TNC+TUDCA for 72 h. Relative quantification of *Il-1β* (**A**), *Cox2* (**B**), *Il-6* (**C**), and *Il-8* (**D**), expressions in normoxic and hypoxic conditions. Results of qRT-PCR are shown as relative fold change in mRNA expression in comparison to untreated controls, where expression level was set as 1. Caspase 1 activity in normoxia and hypoxia (**E**). Results presented as relative luminescence units and compared to untreated controls. Results of qPCR and luminescence-based analyses are presented as mean ± SD from three independent experiments run in triplicate. Significant changes are expressed relative to controls and marked with asterisks and hashes placed above the columns, or as specifically indicated with dashes linking compared treatments. Statistical significance was considered if * *p* < 0.05 or # *p* < 0.001.

Given the high amount of reports informing that excessive and persistent inflammation and cellular stresses are the hallmarks of OA pathology [1,2,6,7,56], we sought to determine whether the applied treatments could modulate inflammatory response in NHAC-kn cells. Therefore, to mimic conditions existing in osteoarthritic chondrocytes, cells were treated with IL-1β and TNC and results evaluated in experimental groups as indicated before (IL-1β, TUDCA, IL-1β+TUDCA, IL-1β+HA, IL-1β+HA+TUDCA, TNC, and TNC+TUDCA) (Figure 4). To this end, expression of the pro-inflammatory mediators *Il-1β*, *Il-6*, *Il-8,* and *Cox2* was assayed (Figure 4A–D) along with inflammasome formation (Figure 4E). Inflammasome formation was determined using the Caspase-Glo 1 inflammasome assay, while *Il-1β*, *Il-6*, *Il-8,* and *Cox2* expressions were analyzed by qRT-PCR. Results showed that in either hypoxia and normoxia, mRNA expression of *Il-1β* was markedly up-regulated in all treatment variants where IL-1β was added to culture media (Figure 4A). However, only in normoxic conditions, TUDCA and HA were able to significantly reduce the expression of *Il-1β* in comparison to IL-1β only-treated chondrocytes. No such tendency was observed in hypoxic conditions (Figure 4A). Corresponding effects were observed for *Cox2* expression (Figure 4B). Likewise, *Il-6* expression presented similar pattern of gene expression with significant up-regulation of *Il-6* in all IL-1β-treated groups and down-regulation in TNC and TNC+TUDCA-stimulated cells in both hypoxia and normoxia (Figure 4C). In case of both treatment conditions, IL-1β+TUDCA, IL-1β+HA, and IL-1β+HA+TUDCA groups showed markedly reduced expression of *Il-6* in comparison to chondrocytes treated with IL-1β alone (Figure 4C). Additionally, the results obtained for *Il-8* expression are in line with the other tested mediators of inflammation. Here, in both hypoxia and normoxia, pronounced overexpression of *Il-8* was observed in all IL-1β-treated cells, and significant reduction in *Il-8* expression in comparison to IL-1β-stimulated cells was shown in IL-1β+TUDCA, IL-1β+HA, and IL-1β+HA+TUDCA groups (Figure 4D). In contrast, TNC treatment seemed to decrease *Il-8* expression in both conditions (Figure 4D). Altogether, these results indicate that TUDCA is effective in reducing pro-inflammatory response in IL-1β-treated cells. Interestingly, a common expression pattern was observed in terms of all genes. In all cases, increases in gene up-regulation reached higher values in normoxic conditions than were detected in hypoxic conditions (Figure 4A–D). This may suggest the existence of some resistance mechanism enabling better adaptation of hypoxic chondrocytes to unfavorable pro-inflammatory conditions. 

To investigate whether the observed transcriptional changes in *Il-1β*, *Il-6*, *Il-8,* and *Cox2* were accompanied by inflammasome formation, we assessed levels of active caspase 1. Inflammasomes are multiprotein complexes, of which caspase 1 is an essential component, responsible for cleavage of IL-1β and IL-18 [57]. Despite observation of highly increased levels of *Il-1β*, *Il-6*, *Il-8,* and *Cox2*, caspase 1 activity following the indicated treatments failed to reach statistical significance (Figure 4E). These data suggest that although IL-1β treatment provokes pro-inflammatory responses in NHAC-kn cells, no direct activation of inflammasomes seems to be involved in this process.

### 2.5. Analysis of ROS Production

**Figure 5:**
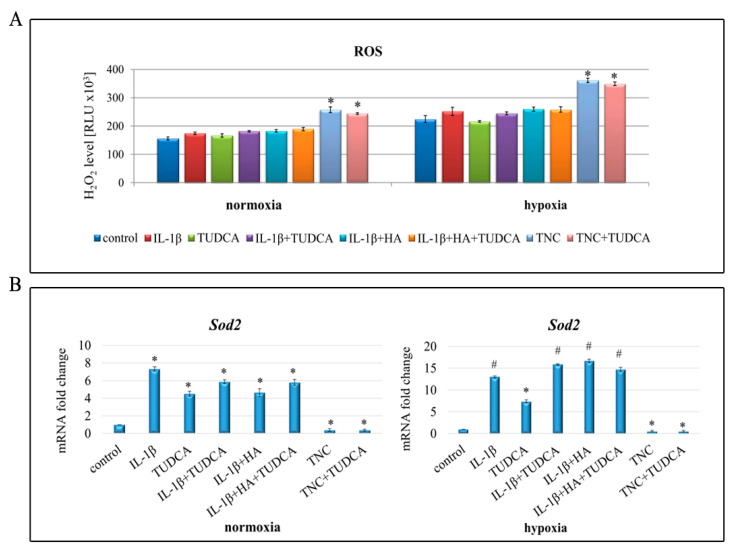

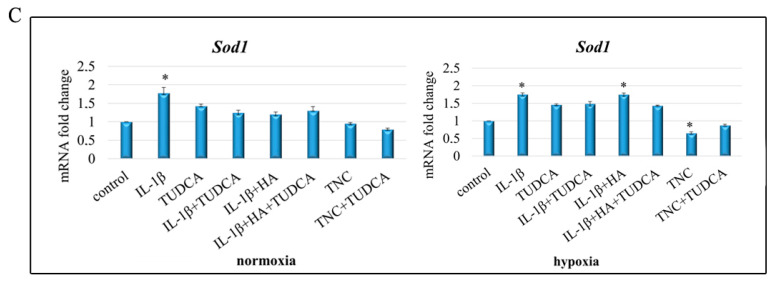
Influence on oxidative stress in NHAC-kn chondrocyte cells stimulated with IL-1β, TUDCA, IL-1β+TUDCA, IL-1β+HA, IL-1β+HA+TUDCA, TNC, and TNC+TUDCA for 72 h. H_2_O_2_ production (**A**). Results presented as relative luminescence units and compared to untreated controls. Relative quantification of *Sod2* (**B**) and *Sod1* (**C**) expressions in normoxic and hypoxic conditions. Results shown as relative fold change in mRNA expression in comparison to untreated controls, where expression level was set as 1. Results of qPCR and luminescence-based analyses are presented as mean ± SD from three independent experiments run in triplicate. Significant changes are expressed relative to controls and marked with asterisks and hashes. Statistical significance was considered if * *p* < 0.05 or # *p* < 0.001.

To gain further insight into the mechanism of IL-1β and TNC toxicity in NHAC-kn chondrocytes, ROS levels were examined (Figure 5A). Thus H_2_O_2_ levels were measured as a convenient proxy for assaying overall ROS levels in cells [57]. Our results demonstrated that in both normoxic and hypoxic conditions, intracellular ROS generation was altered in TNC-treated cells (Figure 5A). TNC caused significant increase in H_2_O_2_ production in normoxic as well as hypoxic chondrocytes; however, we did not manage to fully confirm ROS-defeating properties of TUDCA (Figure 5A). Nevertheless, since it has been known that overproduction of ROS disturbs the balance between oxidative and antioxidative systems resulting in reduced antioxidative capacity, we determined whether enhanced ROS production was accompanied by deregulated expression of key antioxidative enzymes. In this respect, qRT-PCR analysis of *Sod2* and *Sod1* was performed (Figure 5B,C). Indeed, we noticed markedly disrupted expression of the analyzed genes. *Sod2* transcript was intensely up-regulated in IL-1β, TUDCA, IL-1β+TUDCA, IL-1β+HA, and IL-1β+HA+TUDCA treated cells and down-regulated in TNC and TNC+TUDCA groups, in both normoxia and hypoxia (Figure 5B). Interestingly, *Sod1* expression was influenced by all the applied treatments to a lesser extent than *Sod2*. In normoxia, only stimulation with IL-1β resulted in marked overexpression of *Sod1* (1.5-fold increase in comparison to control), while in hypoxia, *Sod1* was overexpressed in IL-1β and IL-1β+HA groups but down-regulated in TNC-treated cells (Figure 5C). These results suggest that treatment with TNC may induce ROS overproduction followed by imbalance of the oxidant–antioxidant system. However, TUDCA was ineffective in diminishing ROS levels in a way to be considered as an antagonist of oxidative stress.

### 2.6. Analysis of Collagen Production

**Figure 6:**
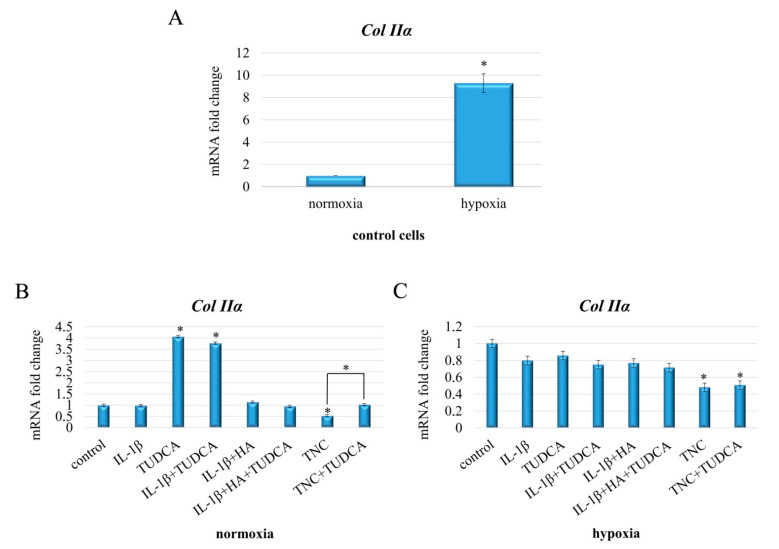
Expression of type II collagen evaluated in NHAC-kn chondrocyte cells stimulated with IL-1β, TUDCA, IL-1β+TUDCA, IL-1β+HA, IL-1β+HA+TUDCA, TNC, and TNC+TUDCA for 72 h. Relative quantification of *Col IIα* expression in normoxic vs. hypoxic control cells (**A**). Relative quantification of *Col IIα* expression in cells subjected to indicated treatments in normoxic (**B**) and hypoxic (**C**) conditions. Results of qPCR analysis are presented as mean ± SD from three independent experiments run in triplicate. Significant changes are expressed relative to controls and marked with asterisks and hashes placed above the columns, or as specifically indicated with dashes linking compared treatments. Statistical significance was considered if * *p* < 0.05.

Next, we investigated the effect of the indicated treatments (IL-1β, TUDCA, IL-1β+TUDCA, IL-1β+HA, IL-1β+HA+TUDCA, TNC, and TNC+TUDCA) on collagen type II expression in NHAC-kn chondrocyte cells (Figure 6). It was measured using qRT-PCR (Figure 6A–C). Since it is already known that sustained hypoxia enhances chondrocyte matrix synthesis [58,59], first, we decided to measure *Col IIα* expression in control normoxic cells vs. control hypoxic cells. Indeed, as shown in Figure 6A, basal expression of *Col IIα* in control cells was significantly affected by hypoxic conditions, presenting over nine-fold up-regulation in comparison to normoxic cells. Subsequently, all the above mentioned treatments were tested in terms of the influence on *Col IIα* expression. 

Interestingly, normoxia treatment with IL-1β did not significantly down-regulate *Col IIα*. However, *Col IIα* expression was markedly up-regulated in cells treated with TUDCA and IL-1β+TUDCA but not in chondrocytes cultured with IL-1β+HA and IL-1β+HA+TUDCA in comparison to control (Figure 6B). Additionally, stimulation with TNC resulted in marked down-regulation of *Col IIα*, and its expression was restored to control levels in TNC+TUDCA cells (Figure 6B). Opposite effects were observed in hypoxic conditions. Here, cells stimulated with IL-1β, TUDCA, IL-1β+TUDCA, IL-1β+HA, and IL-1β+HA+TUDCA showed attenuated, although statistically insignificant, expression of *Col IIα*, and this effect was more pronounced and statistically relevant in TNC and TNC+TUDCA treated cells (Figure 6C). Furthermore, neither TUDCA nor HA were effective in restoring *Col IIα* expression to control level in cells stimulated with either IL-1β or TNC (Figure 6B). Altogether, our results confirmed that TUDCA can enhance *Col IIα* expression in normoxic cells, but failed to clearly demonstrate its positive influence on the expression of type II collagen in hypoxia. Thus, since there are significant differences in response to applied treatments between normoxic and hypoxic cells, the impact of experimental conditions should always be considered when evaluating the influence of pharmacological agents on synthesis of ECM components in chondrocytes.

### 2.7. Analysis of Membrane Surface Charge Density

**Figure 7:**
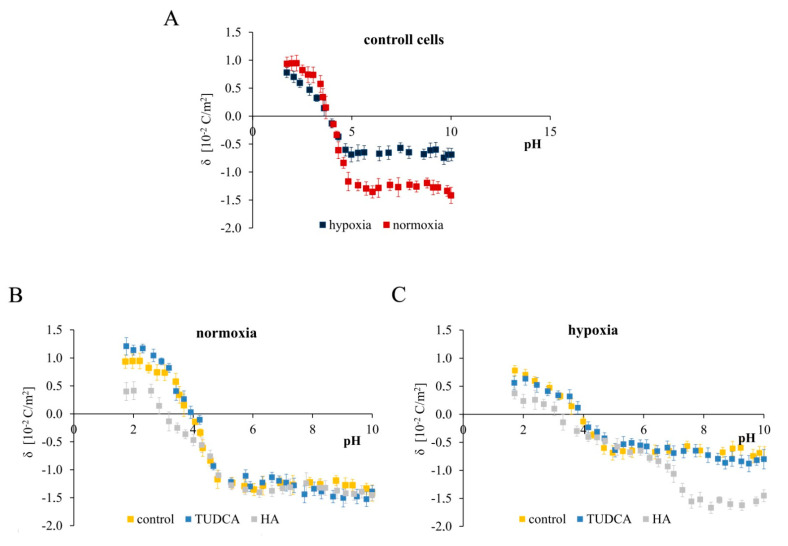
Typical pH-dependence of the surface charge density of NHAC-kn cell membranes. Results for untreated control cells cultured in normoxia vs. hypoxia (**A**), and control cells vs. cells treated with TUDCA and HA in normoxic (**B**) and hypoxic (**C**) conditions are shown.

According to the newest results, biological membranes of chondrocyte cells might be important factors mediating the cellular response to TUDCA treatment [33]. It has been demonstrated that TUDCA caused significant reduction in levels of intracellular cholesterol, augmented membrane fluidity, and increased stability of focal adhesion proteins in chondrocyte cells [33]. Considering the fact that different response to TUDCA and HA treatments were obtained dependently on culture conditions (normoxia vs. hypoxia), we decided to measure surface charge densities of control NHAC-kn cells and those treated with TUDCA (300 µM) and HA (800 µg/mL) in both experimental conditions to get fuller insight into the mechanism of these differences. The pH dependencies of the surface charge of the NHAC-kn cell membranes are plotted in Figure 7. Data are presented for untreated control chondrocytes (Figure 7A) as well as cells treated with TUDCA and HA in normoxic (Figure 7B) and hypoxic (Figure 7C) conditions. The obtained dependencies are of similar shape for all analyzed cell membranes. The decrease in pH values was followed by an increase in positive surface charge density, but only up to a certain point. Conversely, along with an increase in pH values, the negative charge of the membranes increased until the plateau was achieved. 

As shown in Figure 7A, there are visible differences between the surface charge densities values of the membranes in whole analyzed pH range in normoxic and hypoxic conditions. At low pH values, the higher positive surface charge was obtained for membranes in normoxia, whereas lower values were observed for membranes in hypoxia. At high pH values, the higher negative surface charge was obtained in normoxic conditions, while the lower ones were present in hypoxia. Of note, no visible changes were observed in the isoelectric point values in chondrocyte cell membranes in normoxia or hypoxia. The dependencies plotted in the Figure 7B show that for low pH values, the presence of TUDCA caused only slight increase in the positive surface charge density, while the presence of HA resulted in marked decrease in the positive surface charge density. On the other hand, for higher pH values (from approx. 4.2), no significant influence of both acids on surface charge of the chondrocyte membranes was observed. Moreover, HA also significantly influenced the shift in isoelectric point of membranes, from pH ~ 3.9 to pH ~3.0. Figure 7C shows the microelectrophoretic data obtained for cells in hypoxia conditions. As displayed in Figure 7C, the curves obtained for the control and TUDCA-treated cells almost overlap, which indicates that TUDCA does not change the physicochemical properties of the chondrocyte membranes within the entire tested pH range. This allows the conclusion that within a wide range of pH, TUDCA does not adsorb on the membrane surface and easily passes through the lipid bilayer to act inside the cell. On the other hand, HA caused a marked decrease in the positive charge in low pH values and a huge increase in the negative charge for high pH values. It also shifted the isoelectric point towards the more acidic side again, and this shift was similar to the one observed in normoxia. Overall, it can be concluded that HA adsorbs partially or completely on chondrocyte membranes in both normoxic as well as hypoxic cells. However, the changes caused by HA varied, which might be caused by the difference in type and the amount of functional groups located on the surface of cell membranes in both experimental conditions. 

According to these results, biological membranes might be important modulators of cell–drug interactions. Additionally, the differences between normoxic and hypoxic control chondrocytes may result not only from molecular intracellular signaling, but also from modulation of the structure of biological membranes. These results warrant further exploration of membrane engagement in chondrocyte functioning. 

## 3. Discussion

Despite the plethora of data displaying promising results of chemical chaperones in model studies [23,32,60,61,62], only a restricted amount of research reports their influence on chondrocyte cells [6,22,33,63]. In this respect, here we decided to check if the chemical chaperone TUDCA might be effective in restoring proper functioning of chondrocytes subjected to IL-1β and TNC treatment. We first evaluated the influence of IL-1β and TNC as well as TUDCA and HA on cell viability of NHAC-kn cells. As expected, IL-1β and TNC caused a significant decrease in viability of chondrocyte cells, which is in line with numerous other reports [6,11,43,47]. Additionally, neither TUDCA nor HA were cytotoxic to chondrocytes even in high concentrations, which is also in agreement with already available data [6,33,34]. In order to best mimic the conditions present in osteoarthritic chondrocytes, we conducted our research using IL-1β as pro-inflammatory stimulator and TNC to trigger ER stress. Additionally, cells were cultured in normoxic and hypoxic conditions. We observed that despite reduced cell viability caused by IL-1β stimulation, IL-1β did not manage to evoke apoptosis of NHAC-kn chondrocytes. This may be explained by the fact that apoptosis is not exclusively responsible for reduced cell viability. In fact, other phenomena such as cell cycle arrest and premature senescence have been suggested to underlie IL-1β-induced cytotoxicity in chondrocytes [64]. However, cells treated with TNC resulted in markedly augmented apoptotic cell death. Noteworthily, TUDCA failed to rescue cells from TNC-mediated apoptosis in either hypoxia or normoxia. Our results are in partial accordance with the results of Arai et al., who did not observe any relevant reduction in apoptotic cells between TUDCA-treated degenerated chondrocytes and degenerated chondrocytes alone [33]. More promising results have been achieved by Liu et al., who demonstrated that TUDCA enhanced proliferation and reduced apoptosis of TNC-treated chondrocytes by approximately 18% [6]. However, in this study, apoptosis was evaluated after nine days of treatment. Notably, up to the fifth day of incubation, the effect of TUDCA on apoptosis was insignificant, which may explain the discrepancies between our results.

Furthermore, to investigate if the pro-apoptotic effect was at least partially mediated by ER stress, we analyzed the expression of several molecular markers of ER stress on the transcriptomic and proteomic level. As predicted, treatment with TNC resulted in significant elevation of ER stress in chondrocyte cells, as evidenced by augmented levels of GRP78, GRP94, ORP150/170, and CHOP expressions. Additionally, TNC-derived effects seemed to be independent on culture conditions being similar in normoxia and hypoxia. Interestingly, although TUDCA markedly decreased the expression of CHOP in TNC-stimulated cells, this did not contribute to reduction of apoptosis in NHAC-kn chondrocytes. This may suggest the existence of other pro-apoptotic pathways activated in addition to ER stress after exposition to TNC. Indeed, overproduction of ROS has been already demonstrated to contribute to apoptosis of cardiomyocytes and cardiomyoblasts after TNC treatment [48,49]. In our study we also found a small but significant increase in ROS generation in TNC-treated cells, which was not affected by TUDCA supplementation. This partially explains the unchanged apoptosis ratio in TNC-treated cells co-cultured with TUDCA. Unfortunately, there is still a limited amount of research concerning this issue on chondrocytes. Nevertheless, the results of Liu et al., who demonstrated decreased expression of Bcl-2 and overexpression of Bax and caspase 9 in chondrocytes exposed to TNC, may suggest involvement of intrinsic apoptotic pathway in death of TNC-stimulated chondrocytes [63]. This seems to be in line with our results showing pronounced down-regulation of *Sod2* in TNC-treated cells, which was not further restored by TUDCA. Since SOD2 is a mitochondrial antioxidant enzyme, we speculate that its reduced expression is likely due to failed mitochondria efforts to overcome TNC-mediated mitochondria imbalance, which resulted in ROS overproduction. 

Interestingly, treatment with IL-1β also disrupted ER homeostasis, as evidenced by increased GRP78/GRP94 expression, however only in normoxic cells. In hypoxia, GRP78/GRP94 levels in control cells were already overexpressed in comparison to control cells cultured in normoxic conditions. Nevertheless, co-treatment with both TUDCA and HA significantly decreased expression of these chaperone proteins in comparison to stimulation with IL-1β alone, which is also in partial agreement with previous reports [8,22,27]. Husa et al. demonstrated that IL-1β signaling can stimulate UPR activation in chondrocytes [27]. It has been shown that IL-1β elevated the expression of GRP78, however without activating CHOP, which is also in line with our results [27]. This may confirm that TUDCA may be efficient in reduction of mild ER stress caused by IL-1β in chondrocytes. Additionally, although demonstrated in previous studies [43,46], our results showed that stimulation with IL-1β did not evoke relevant elevation in ROS levels. However, chondrocytes treated with either IL-1β, TUDCA, IL-1β+TUDCA, IL-1β+HA, and IL-1β+HA+TUDCA in both normoxia and hypoxia showed drastic increase in *Sod2* expression. Thus, intact ROS levels may be explained by strong overcompensation of IL-1β-induced ROS generation by antioxidant response in affected chondrocytes. Our results are in line with the findings of Khan et al., who reported that chondrocytes treated with IL-1β showed marked Nrf2/ARE-dependent overexpression of antioxidant enzymes such as HO-1, NQO1, and SOD2 [45]. Importantly, they also found that IL-1β treatment resulted in little to no induction of ROS in normal chondrocytes, while leading to significantly elevated ROS levels in osteoarthritic chondrocytes [45]. Together with our results, this confirms that normal chondrocytes can withstand the exposition to IL-1β and prevent the development of oxidative stress, most probably due to augmented efforts from the antioxidant system. 

One of the most known effects of IL-1β stimulation in chondrocytes is triggering the pro-inflammatory response [42,43,44]. Indeed, IL-1β-mediated overexpression of IL-1β, TNF-α, IL-6, IL-8, and COX2 was already demonstrated in various studies [42,43,44]. Our results seem to be in alignment with previous reports, as we also demonstrated markedly up-regulated expressions of *Il-1β*, *Il-6*, *Il-8,* and *Cox2* in IL-1β-stimulated NHAC-kn cells, but not in TNC-treated cells. Notably, a similar pattern of expression showing significant down-regulation of all tested genes in cells co-treated with TUDCA and HA was visible in normoxic as well as hypoxic conditions. These results seem to be in agreement with other reports demonstrating the anti-inflammatory effect of TUDCA [32]. Although to date there are no results demonstrating such an effect of TUDCA in chondrocyte cells, this activity has already been confirmed in models of other diseases such as asthma, hepatic ischemia reperfusion, or neurological disorders [32]. However, the role of HA in alleviation of inflammation can be controversial considering the possibility of only partial HA intracellular penetration due to its high molecular weight [35,36,37,38,39]. Indeed, since TUDCA was demonstrated to cross cellular membrane with no restrictions, ξ-potential analysis revealed that HA altered surface charge density of cellular membranes, suggesting at least partial adsorption on chondrocytes’ membranes and limited cellular intake. Thus, the IL-1β-contradicting effect of HA may be due to its external functioning. Indeed, studies have shown that HA may prevent binding of IL-1β to its membrane-anchored receptor by covering the cell surface and thereby inhibiting IL-1β-dependent effects [65,66]. Surprisingly, in hypoxia, cells stimulated with TUDCA alone showed small but relevant up-regulation of almost all tested pro-inflammatory mediators, which may seem contradictory to the down-regulating effect of TUDCA observed in cells co-treated with IL-1β. One possible explanation of this phenomenon might be connected with the effect of hypoxic conditions per se and the up-regulation of hypoxia inducible factors. It has been demonstrated that hypoxia led to the activation of HIF2α, which was involved in the activation of pro-inflammatory cytokines in mice model of rheumatoid arthritis [67]. Additionally, HIF1α was shown to mediate transcriptional activation of IL-1β in astrocyte cultures [68]. Nevertheless, this issue needs further investigation to fully explain mechanisms triggering molecular response of chondrocytes to TUDCA, especially in hypoxic conditions. Notably, despite pronounced overexpression of many pro-inflammatory mediators, we did not identify increased activity of caspase 1 either in hypoxic or normoxic conditions, suggesting no direct activation of inflammasomes.

Finally, we examined how TUDCA co-treatment would influence collagen II expression in chondrocytes exposed to IL-1β and TNC in normoxic and hypoxic conditions. It has already been well documented that collagen II expression is strongly dependent on oxygen levels, presenting marked overexpression of this protein in hypoxic conditions [42,44,63,69,70]. Additionally, both inflammation and ER stress have been shown to down-regulate collagen II in chondrocytes [42,44,63,69,70]. In line with these results, we also demonstrated marked increase in *Col IIα* expression in hypoxic cells in comparison to normoxic counterparts. However, TUDCA was only able to restore TNC-diminished levels of *Col IIα* in normoxic chondrocytes, and seemed to influence *Col IIα* expression to a lower extent in hypoxia. Intuitively, this might seem reasonable, since hypoxia per se is a well-known inducer of collagen II production in chondrocytes, and further TUDCA-dependent overstimulation of *Coll II* expression might seem unnecessary and aggravating to cells. Of note, in normoxia, TUDCA alone and in co-treatment with IL-1β potentiated the expression of *Col IIα* in comparison to control and IL-1β-stimulated cells. These results are in partial agreement with previous reports demonstrating the stimulating effect of TUDCA on collagen II expression in TNC-treated and degenerated chondrocytes [22,33]. Nevertheless, these results are only partially promising, since up-regulation of *Col IIα* failed to be confirmed in hypoxic conditions. Interestingly, concerning 21% of oxygen being excessive for chondrocytes and a limiting factor for collagen II production, perhaps TUDCA might serve as an alternative stimulator of *Coll II* expression in unfavorable oxygen conditions. These discrepancies demonstrate that many conditions can influence chondrocyte functioning and numerous factors should be considered in designing future chondrocyte-based studies. Especially, more complex examinations mirroring actual oxygen conditions present in cartilage (≈1% O_2_ for hypoxia and ≈5% O_2_ for normoxia) should be further performed to untangle the complicated nature of chondrocytes’ response to pharmacological treatments.

## 4. Materials and Methods

### 4.1. Reagents

CGMTM Chondrocyte Growth BulletKitTM containing fetal bovine serum (FBS), GA-1000, R3-IGF, bFGF, transferrin, and insulin were provided by Lonza (Walkersville, MD, USA). A high-capacity RNA-to-cDNA kit was purchased from Thermo Fisher Scientific (Waltham, MA, USA). The ReliaPrep RNA Cell Miniprep system, ROS-Glo H_2_O_2_ assay, Caspase-Glo 3/7 assay, and Caspase-Glo 1 inflammasome assay were provided by Promega (Fitchburg, WI, USA). A radioimmunoprecipitation-assay (RIPA) lysis buffer and BCA protein-assay kit were from Thermo Fisher Scientific. SigmaFast BCIP/NBT reagent and molecular-grade purity water were provided by Sigma-Aldrich (St Louis, MO, USA). The polyclonal (mouse) anti-KDEL antibody was purchased from Enzo Biochem (Farmingdale, NY, USA) and monoclonal (mouse) anti-HIF1α antibody from BD Biosciences (San Jose, CA, USA). Horseradish peroxidase (HRP)-conjugated anti-mouse IgG was from Rockland Immunochemicals (Limerick, PA, USA). HRP-conjugated anti-rabbit IgG antibody, polyclonal (rabbit) anti-β-tubulin antibody, monoclonal (mouse) anti-CHOP antibody, monoclonal (mouse) anti-XBP-1s antibody, polyclonal (rabbit) anti-ORP150 antibody, and SignalFire Elite ECL Reagent were provided by Cell Signaling Technology (Boston, MA, USA). Tauroursodeoxycholic acid (TUDCA), hyaluronic acid (HA), and tunicamycin (TNC) were purchased from EMD Millipore corporation (Temecula, CA, USA). 

### 4.2. Cell Culture and Induction of Hypoxia

The normal human knee articular chondrocytes (NHAC-kn) were purchased from Lonza (Walkersville, MD, USA). Cells were cultured in a specifically dedicated Chondrocyte Growth Medium (CGMTM) with the de-differentiation preventing supplement containing 0.2% R3-IGF-1, 0.5% rhFGF, 0.1% transferrin, 0.2% insulin, 5% fetal bovine serum (FBS), and 0.1% GA-1000 (gentamicin sulphate + amphotericin B), purchased from Lonza. Normoxic conditions were maintained by culturing cells in Falcon flasks (BD) in a 5% CO_2_ incubator Galaxy S+ (RS Biotech, Irvine, UK), at 37 °C. Hypoxia was evoked by incubation of cells in an atmosphere containing reduced concentration of oxygen (1% O_2_) in hypoxia chamber Galaxy 170R (Eppendorf Inc., Hamburg, Germany). Subconfluent cultures were detached from the flask surface with 0.05% trypsin 0.02% EDTA in calcium-free phosphate-buffered saline (PBS) and counted in cell counter Scepter (Millipore).

### 4.3. Cell Viability

The viability of NHAC-kn cells was evaluated according to method described in Carmichael et al. using 3-(4,5-dimethylthiazol-2-yl)-2,5-diphenyltetrazolium bromide (MTT) [71]. Briefly, cells were seeded in 24-well plates at a density of 5 × 10^4^ cells/well. Confluent cells were then cultured with a set of substances (TUDCA, HA, IL-1β, TNC) in a wide range of concentrations: IL-1β (5–40 ng/mL), TUDCA (50–1500 µM), HA (100–1500 µg/mL), and TNC (0.25–7 µg/mL) for 24, 48, and 72 h. Next, cells were washed twice with PBS and incubated with 1 mL MTT solution (0.25 mg/mL in PBS) at 37 °C in a humidified 5% CO_2_ atmosphere for 3 h. The medium was removed and formazan products solubilized in 1 mL of 0.1 mmol/L HCl in absolute isopropanol. Absorbance of a converted dye in living cells was read on a microplate reader (Tecan, Männedorf, Switzerland) at a wavelength of 570 nm. The viability of NHAC-kn cells was calculated as a percentage of control untreated cells. 

### 4.4. Cell Morphological Analysis

To visualize morphological characteristic of chondrocytes, NHAC-kn cells were exposed to various treatments (control, IL-1β, TUDCA, IL-1β+TUDCA, IL-1β+HA, IL-1β+HA+TUDCA, TNC, TNC+TUDCA) and kept in normoxic and hypoxic conditions for 72 h. Then, cells were observed under phase-contrast inverted microscope. Images of chondrocytes were captured using a CKX 41 microscope (Olympus, Tokyo, Japan) at 100× magnification. No specific staining was carried out.

### 4.5. Detection of Apoptosis

Measurement of caspase 3/7 activity after treatment with IL-1β, TUDCA, IL-1β+TUDCA, IL-1β+HA, IL-1β+HA+TUDCA, TNC, TNC+TUDCA was performed using the luminescent Caspase-Glo 3/7 assay following the manufacturer’s instructions. Briefly, NHAC-kn cells were seeded in white-walled 96-well culture plates (Nunclon; Thermo Fisher Scientific) at a density of 1 × 10^3^ cells/well. Subsequently, cells were incubated with medium containing all the above-mentioned combinations of tested agents (at concentrations selected on the basis of cell viability results as indicated in the Section 2) for 72 h. After incubation, 100 μL of Caspase-Glo 3/7 reagent was added to each sample. Cells were mixed using a plate shaker at 300 rpm for 45 s and left in the dark at room temperature for 40 min. This was followed by measurement of luminescence with a microplate reader (Tecan).

### 4.6. RNA Isolation and Gene Expression Analysis

Total RNA was isolated using the ReliaPrep system with DNase I treatment according to the manufacturer’s instructions. Spectrophotometric measurements were performed to evaluate the quality and quantity of the extracted RNA (NanoPhotometer; Implen, Munich, Germany). Synthesis of cDNA was performed using the high-capacity RNA-to-cDNA Kit following the supplier’s recommendations. Briefly, 0.5 μg of purified total RNA was used in a 20 μL of reaction mixture containing oligo(dT)16 primers, random octamers, dNTPs, and murine leukemia virus reverse transcriptase (RT). cDNA (2 μL) served as a template for real-time RT quantitative polymerase chain reaction (qPCR). Amplification of the product was performed using 2×HS-PCR Master Mix SYBR A (A&A Biotechnology, Gdynia, Poland). Primer sequences for *Chop*, *Grp78*, *Sod1*, *Sod2*, *Cox2*, *IL-1β,* and housekeeping *Rpl13a* have been featured in our previous work [57]. Sequences of the other PCR primers were previously described as: *ColIIα*, *Xbp1*, and *Il-6* [17]; *Hif1α* and *Il-8* [72]; and *Hyou1 (Orp150)* [73]. Additional evaluation of primer accuracy was done using Primer-BLAST software (https://www.ncbi.nlm.nih.gov/tools/primer-blast/, accessed on 30 December 2020). The following reaction parameters were applied in a thermal cycler: initial denaturation at 95 °C for 3 min, followed by 40 cycles of 95 °C for 1 min, 60–69 °C for 30 s, and 72 °C for 45 s. The CFX Connect real-time PCR system (Bio-Rad Laboratories, Hercules, CA, USA) was used to perform a real-time qPCR assay. Reactions were run in triplicates and expressions were analyzed using the relative quantification method modified by Pfaffl [74].

### 4.7. Protein Assay

NHAC-kn cells were seeded in 10 cm culture dishes and treated as previously described. After treatment, cells were washed with cold PBS and solubilized in 200 μL RIPA lysis buffer per well. Cell lysates were then subjected to centrifugation (14,000× *g* at 4 °C for 10 min), and supernatants were collected for protein evaluation. The BCA protein-assay kit was used to determine protein concentration in cell lysates. Protein assays were performed according to the method described by Smith et al. [75]. Bovine albumin was used as a standard.

### 4.8. Sodium Dodecyl Sulfate–Polyacrylamide-Gel Electrophoresis

Samples of the lysates containing 20 μg of protein were subjected to sodium dodecyl sulfate–polyacrylamide-gel electrophoresis as described by Laemmli [76]. Electrophoresis was run for 40–45 min using a 10–12% polyacrylamide-gel. A constant current of 25 mA was applied.

### 4.9. Immunoblotting

Western blot analysis was performed in order to validate qPCR results. In this respect, resolved proteins were transferred to polyvinylidene difluoride (PVDF) membranes and preincubated with Tris-buffered saline (TBS) containing 0.05% Tween 20 (TBS-T) and 5% nonfat dry milk for 2 h. Membranes were soaked in a mixture of polyclonal (mouse) anti-KDEL antibody (1:1000) and polyclonal (rabbit) anti-β-tubulin antibody (1:1000), monoclonal (mouse) anti-CHOP antibody (1:1000), monoclonal (mouse) anti-XBP-1s antibody (1:1000), polyclonal (rabbit) anti-ORP150 antibody (1:1000), and monoclonal (mouse) anti-HIF1α (1:500) in 5% dried milk in TBS-T at 4 °C for 16 h. Next, 1-h incubation with secondary HRP-conjugated antibody against mouse or rabbit IgG at 1:2500 dilution was carried out. Finally, the PVDF membranes were washed five times with TBS-T and exposed to SignalFire Elite ECL Reagent (Cell Signalling). Images were visualized using GeneGnome XRQ Chemiluminescence system (Syngen, Cambridge, UK). 

### 4.10. Reactive Oxygen Species Generation

Generation of ROS was detected using the luminescent ROS-Glo H_2_O_2_ assay. NHAC-kn cells were plated at a density of 1 × 10^3^ per well in 80 μL of CGM medium in 96-well white-walled plates (Nunclon), as recommended by the manufacturer. Briefly, cells were allowed to attach to the plates at 37 °C in a CO_2_ incubator, and then growth medium was replaced with CGM medium containing IL-1β, TUDCA, IL-1β+TUDCA, IL-1β+HA, IL-1β+HA+TUDCA, TNC, and TNC+TUDCA and grown for 72 h. Substrate solution was added to cells in a final concentration of 25 μmol/mL. Then, the cells were returned to the incubator (5% CO_2_, 37 °C) and cultured for 6 h. After this, 100 μL ROS-Glo detection solution was added to each well for 20 min at room temperature, and then relative luminescence units were recorded using the microplate reader. 

### 4.11. Caspase 1 Activity Assessment

Inflammasome formation was determined using the bioluminescent Caspase-Glo 1 Inflammasome assay. NHAC-kn cells were seeded at 1 × 10^3^ cells/well in 100 μL CGM medium in 96-well white-walled plates according to the manufacturer’s instructions. Briefly, 100 μL Caspase-Glo 1 reagent (containing MG132 inhibitor in the final concentration of 60 μmol/L) or Caspase-Glo 1 YVAD-CHO reagent (containing Ac-YVAD-CHO inhibitor at a final concentration of 1 μmol/L) was added to the 96-well plate containing 100 μL of blank reaction, negative control cells, or treated cells in culture medium. Next, plates were covered with a lid, and well contents were gently mixed using a plate shaker at 300 rpm for 30 s. Plates were incubated at room temperature for 1 h to allow stabilization of the luminescent signal. Luminescence was recorded using the microplate reader.

### 4.12. Zeta Potential Measurements

Mobility of cell membrane was carried out using the electrophoretic light scattering technique on Zetasizer Nano ZS analyzer equipped with a 4 mW He-Ne laser (Malvern Instruments, Malvern, UK). The measurements were performed as a function of pH (in pH range 2–10). Briefly, chondrocytes were suspended in 0.9% NaCl and titrated to the desired pH with HCl or NaOH. The reported values represent the average of six measurements at a given pH value. Based on electrophoretic mobility values, the surface charge density (*δ*) was determined from the equation [1]:

(1) $$\delta \;=\;\frac{\eta \cdot u}{d}$$

in which: *η*—the viscosity of solution, *u*—the electrophoretic mobility, *d*—the diffuse layer thickness.

### 4.13. Statistical Analysis

Results are presented as mean ± SD from three independent experiments run in triplicate. GraphPad Prism 5.0 software (La Jolla, CA, USA) was used to perform statistical analyses. One-way analysis of variance (ANOVA) was carried out for comparisons between control and treated groups. Pairwise comparisons were made by post hoc Tukey’s test. Post hoc tests were run only if *F* achieved the necessary level of statistical significance and there was no significant variance inhomogeneity. Differences were considered significant for * *p* < 0.05 or # *p* < 0.001.

## 5. Conclusions

One big advantage of potential utilization of TUDCA in OA therapy is the route of administration. In case of most diseases, use of TUDCA depends on oral or intravenous administration, which may be controversial regarding systemic effects of this bile acid. Lately, one popular medical procedure improving joint malfunction is direct intra-articular injection of HA, which restores physicochemical properties of synovial fluid and supports proper functioning of joints. Since we demonstrated that HA/TUDCA co-treatment did not cause any negative effects nor markedly alleviate effectiveness of TUDCA alone, this way of administration might be considered as possible treatment option in the future. Moreover, since we showed that TUDCA can easily cross biological membranes and HA most likely retains on the cell surface, these two compounds could complement each other’s action to best serve as potential treatment in osteoarthritic patients. These premises strongly encourage further studies targeted at exploring the role of TUDCA in chondrocytes’ biology. 

Despite growing interest in utilization of TUDCA in treatment of many conformational diseases, still a very limited number of reports exist in terms of its effectiveness in chondroprotection. Although two existing reports suggest promising effects of TUDCA in chondrocytes [6,33], results of our experiments are only partially optimistic. These discrepancies may be explained by many factors including different cell type, medium composition, oxygen conditions, or time of incubation and dose of used TUDCA. Despite managing to demonstrate good chaperoning-like activity of TUDCA in normoxic conditions, we failed to show its efficacy in hypoxia. Exceptionally promising results, however, regarding both culture conditions came from the studies of the anti-inflammatory potential of TUDCA. Marked decrease in main pro-inflammatory mediators, especially *Cox-2*, can make TUDCA a promising alternative treatment/co-treatment in osteoarthritis. Nevertheless, studies should be continued in order to explore the issue of using TUDCA as pharmacotherapeutic agent for OA in the future.

## Figures and Tables

**Table 1:** Pharmacological agents used in the study. Previously reported effects in chondrocytes together with proper references numbers are listed.

Agent	Symbol	Reported Effect	References
**Tauroursodeoxycholic acid**	**TUDCA**	Alleviation of ER stress	[6]
		Alleviation of inflammation	[32] ^1^
		Alleviation of oxidative stress	[32] ^1^
		Restoration of collagen expression	[6,33]
**Hyaluronic acid**	**HA**	Alleviation of oxidative stress	[34,35,36]
		Alleviation of inflammation	[37,38,39]
		Restoration of collagen expression	[40,41]
**Interleukin-1β**	**IL-1β**	Induction of inflammation	[5,42,43,44]
		Apoptosis	[21,22,27,42,43,45]
		Induction of ER stress	[8,17,22,27]
		Induction of oxidative stress	[27,45,46]
		Reduction of collagen expression	[21,22,40,41]
**Tunicamycin**	**TNC**	Induction of ER stress	[6,11,18,27,47]
		Apoptosis	[6,11,18,47]
		Induction of oxidative stress	[48,49] ^1^
		Reduction of collagen expression	[6,18]

^1^ not studied in chondrocytes; reported in other cell types.

## Data Availability

The data presented in this study are available in this manuscript.

## Sample Availability

Samples of the compounds are available from the commercial sources as stated in the Section 4.
